# Capecitabine, 5-fluorouracil and S-1 based regimens for previously untreated advanced oesophagogastric cancer: A network meta-analysis

**DOI:** 10.1038/s41598-017-07750-3

**Published:** 2017-08-02

**Authors:** Emil ter Veer, Lok Lam Ngai, Gert van Valkenhoef, Nadia Haj Mohammad, Maarten C. J. Anderegg, Martijn G. H. van Oijen, Hanneke W. M. van Laarhoven

**Affiliations:** 1 Cancer Centre Amsterdam, Department of Medical Oncology, Academic Medical Centre, University of Amsterdam, Amsterdam, The Netherlands; 2 Department of Epidemiology, University of Groningen, University Medical Centre Groningen, Groningen, The Netherlands; 3 Department of Surgery, Academic Medical Centre, University of Amsterdam, Amsterdam, The Netherlands

## Abstract

As evidence is inconsistent and based on either isolated Asian or Western studies, we conducted a network meta-analysis (NMA) to examine efficacy and safety of 5-FU (5-fluorouracil), capecitabine and S-1-based first-line treatment of advanced esophagogastric cancer in Asian and Western patients. Medline, EMBASE, CENTRAL and conferences ASCO and ESMO were searched up to January 2016 for randomized-controlled-trials comparing 5-FU, capecitabine or S-1-based regimens with equal chemotherapy backbones. Direct and indirect data for overall survival (OS) and progression-free-survival (PFS) were combined on the Hazard Ratio (HR)-scale using random-effects NMA and calculated as combined HRs and 95%credible intervals (95%CrI). Grade 1-2 and grade 3-4 adverse events were compared with pair-wise meta-analysis. Fifteen studies were identified including capecitabine (n = 945), 5-FU (n = 2,132) or S-1 (n = 1,636). No differences were found in respectively OS and PFS for capecitabine-based versus 5-FU-based regimens (HR = 0.89, 95%CrI = 0.76–1.04 and HR = 0.98, 95%CrI = 0.75–1.32), S-1-based versus 5-FU-based regimens (HR = 0.92, 95%CrI = 0.82–1.04 and HR = 0.88, 95%CrI = 0.70–1.11) and S-1-based versus capecitabine-based regimens (HR = 1.03, 95%CrI = 0.87–1.22 and HR = 0.89, 95%CrI = 0.65–1.20). Effects were similar in Asian and Western subgroups. Toxicity profiles were different but a lower frequency of relevant adverse events was observed with S-1 In conclusion, as efficacy was similar, choosing fluoropyrimidines should be based on their individual toxicity profiles.

## Introduction

Advanced esophagogastric adenocarcinoma (AEGC) of the stomach, gastro-esophageal junction (GEJ) or esophagus is one of the major causes of cancer-specific mortality^1^. In the late nineties, it was shown that survival could be extended by palliative chemotherapy^2^. After two decades of clinical studies, fluoropyrimidines are still the cornerstone of first-line treatment for advanced esophagogastric cancer. In addition to 5-fluorouracil (5-FU), also two novel fluoropyrimidine compounds have been introduced in first-line treatment of AEGC: capecitabine and S-1. Capecitabine is more frequently prescribed in Western countries, whereas S-1 is more frequently prescribed in Asian countries. Usually, a fluoropyrimidine is combined with either a platinum agent, a taxane or irinotecan^3, 4^.

Whether 5-FU, capecitabine and S-1 based chemotherapy regimens are equally effective in first-line treatment of AEGC is still under debate^5^. There is evidence that there is no difference in efficacy between the three fluoropyrimidines and that S-1 has a more favourable toxicity profile from a previously conducted pair-wise meta-analysis^6^. However, Asian patients may respond differently to S-1 than Western patients, which might be explained by variations in metabolism^7^. The two novel oral fluoropyrimidines capecitabine and S-1 were compared in only a three small phase II RCTs in an Asian patient population, which makes it difficult to explore the specific benefits of capecitabine-based or S-1 based regimens on a global scale^8–10^.

Furthermore, the differences in efficacy and toxicity between capecitabine-based and 5-FU-based regimens have not been addressed previously with meta-analysis including the newest studies. After completion of the REAL-2 study, which found that capecitabine and 5-FU-based regimens were equally effective^11^, a pooled analysis of the REAL-2 trial and the ML17032 trial^12^ indicated a small benefit in survival of capecitabine-based over 5-FU based regimens^13^. Since then, another RCT on this topic has been conducted with conflicting results^14^. Given the available evidence, differences in efficacy between capecitabine-based and 5-FU-based regimens are difficult to explore by pair-wise analyses alone, and network-meta-analyses (NMA) may be more appropriate.

In NMA, a common comparator is used to estimate indirect relative effects between treatments^15^. Both direct and indirect relative treatment estimations can be combined into a combined effect-size. The benefit of this approach is that the precision of the effect estimate is increased and cross-validated among all available studies^16^. Moreover, in case of a low number of direct head-to-head RCTs, NMA can increase power to detect statistically significant differences. Therefore, we conducted a systematic review with NMA to examine the relative efficacy and safety of 5-FU, capecitabine and S-1 based regimens in first-line treatment of AEGC.

## Results

### Description of the included studies

**Figure 1:**
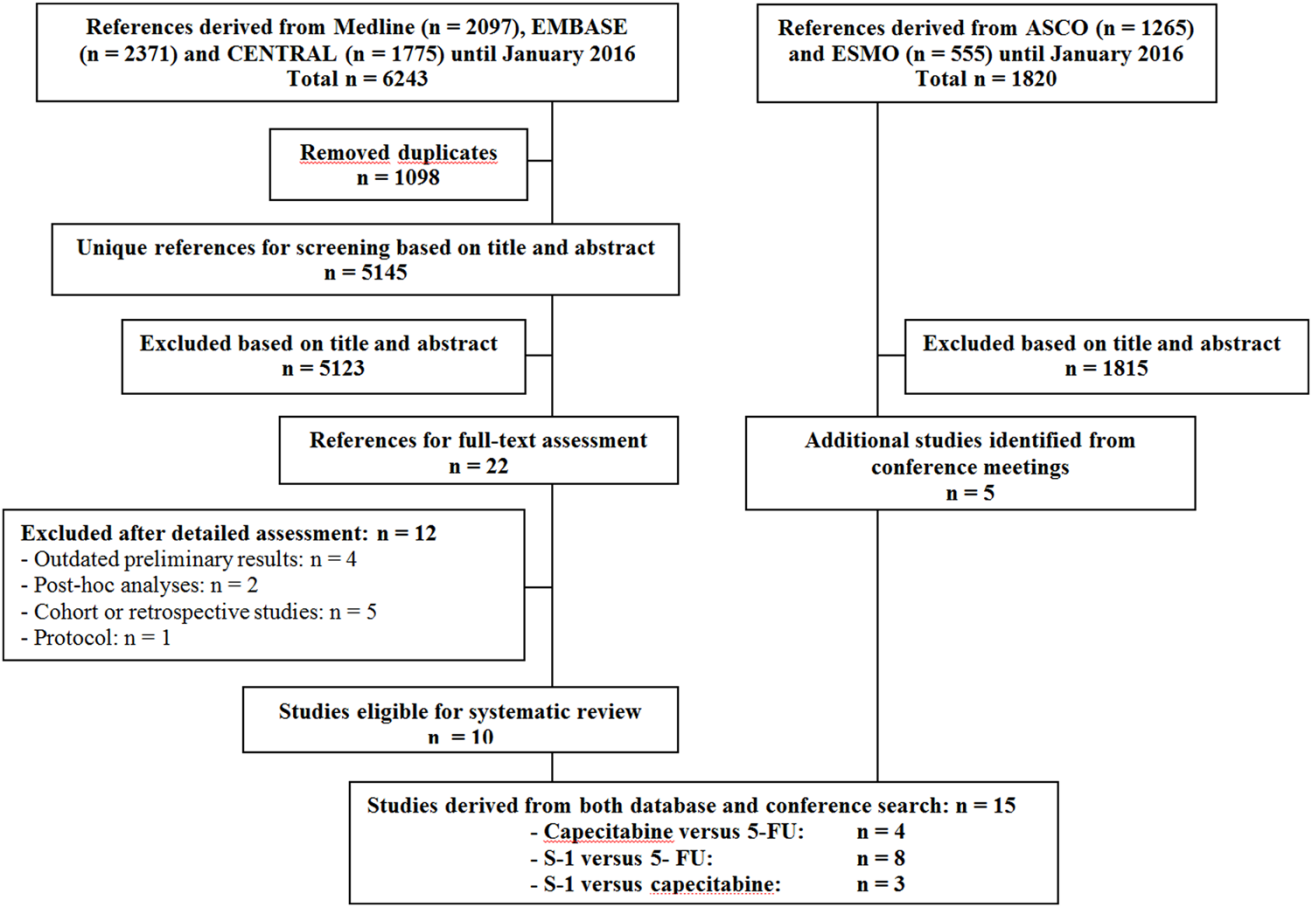
Flowchart of included studies. Flowchart of references derived from database search (left) and from conference search (right).

**Figure 2:**
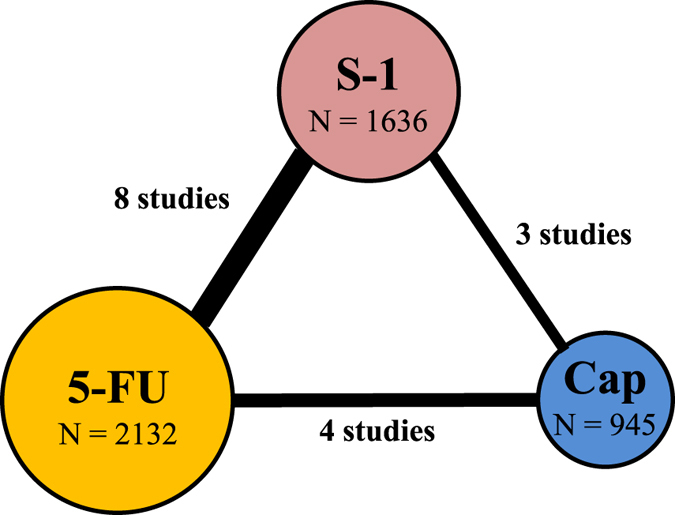
The network meta-analysis. Studies that contained 5-FU, capecitabine or S-1 based regimens with an equal backbone were analysed in a 3-node network. The size of each node corresponds to the number of patients that were randomized to receive S-1 (n = 1636), capecitabine (n = 945) and 5-FU based therapy (n = 2132). The lines connect the regimens that were directly compared in head-to-head RCTs. The thickness of the lines corresponds to the number of RCTs. Abbreviations: 5-FU: 5-fluorouracil.

A total of 5,145 unique titles were retrieved through the database search, of which 22 RCTs remained after screening the titles and abstracts. After full-text assessment, 12 were excluded and ten were found eligible. In addition, five studies retrieved from the conference search were found eligible (Fig. 1). In total, 15 studies containing 4,713 patients were eligible^8–12, 14, 17–25^. The number of patients that received capecitabine-based, 5-FU-based and S-1 based regimens was 945, 2,132 and 1,636 respectively (Fig. 2 and Table 1). HRs for OS and PFS could be extracted in 14 and 9 studies respectively.

**Table 1:** Baseline characteristics.

Study	N	Arms	Ethnicity	Male	Age	Stage IV	WHO ≥ 2	GEJ	Stomach
			Asian or Western	N (%)	median (range)	N (%)	N (%)	N (%)	N (%)
**Capecitabine vs 5-FU**									
Cunningham 2008^11^*	250	Epi + Cis + Cap	Western	201 (81)	64 (25–82)	192 (77)	31 (12)	68 (28)	102 (42)
	244	Epi + Ox + Cap		202 (83)	62 (25–80)	185 (76)	24 (10)	53 (22)	104 (44)
	263	Epi + Cis + 5-FU		214 (81)	65 (22–83)	209 (80)	31 (12)	72 (29)	90 (36)
	245	Epi + Ox + 5-FU		199 (81)	61 (33–78)	189 (77)	21 (9)	55 (23)	87 (37)
Kang 2009^12^	160	Cis + Cap	Asian	103 (64)	56 (26–74)	160 (100)	†16 (10)	0 (0)	160 (100)
	156	Cis + 5-FU		108 (69)	56 (22–73)	156 (100)	†17 (11)	0 (0)	156 (100)
Ocvick 2012^17^	45	Epi + Cis + 5-FU	Western	34 (76)	55 (20–72)	37 (82)	3 (6)	0 (0)	45 (100)
	40	Epi + Cis + Cap		32 (80)	56 (40–77)	35 (88)	2 (5)	0 (0)	40 (100)
Van Cutsem 2015^14^	89	DTX + Ox + 5-FU/Lv	Western	61 (69)	††58	89 (100)	2 (2)	35 (39)	75 (84)
	86	DTX + Ox + Cap		64 (74)	††59	86 (100)	3 (3)	28 (33)	75 (87)
**S-1 vs 5-FU**									
Ajani 2010^18^	521	Cis + S-1	Western	382 (73)	59 (18–83)	497 (96)	0 (0)	82 (16)	438 (84)
	508	Cis + 5-FU		347 (68)	60 (20–85)	488 (96)	0 (0)	88 (17)	417 (82)
Ajani 2015^19^	239	Cis + S-1	Western	124 (52)	56 (25–86)	239 (100)	0 (0)	16 (7)	223 (93)
	122	Cis + 5-FU		60 (49)	56 (27–83)	122 (100)	0 (0)	5 (4)	117 (96)
Boku 2009^20^	234	S-1	Asian	175 (75)	64 (58–69)	234 (100)	3 (1)	0 (0)	234 (100)
	234	5-FU		176 (75)	64 (57–69)	234 (100)	3 (1)	0 (0)	234 (100)
Huang 2013^21^	119	PTX + S-1	Asian	89 (75)	56 (18–74)	112 (94)	Median KPS 80	NA	NA
	110	PTX + 5-FU/Lv		76 (69)	54 (19–72)	102 (93)	Median KPS 80	NA	NA
Jin 2008^22^	74	Cis + S-1	Asian	55 (74)	57 (24–80)	74 (100)	8 (11)	0 (0)	74 (100)
	73	Cis + 5-FU		61 (84)	58 (33–77)	73 (100)	10 (14)	0 (0)	73 (100)
Li 2015^23^	120	Cis + S-1	Asian	84 (70)	53 (25–76)	120 (100)	7 (6)	22 (18)	70 (58)
	118	Cis + 5-FU		85 (73)	55 (21–76)	118 (100)	4 (3)	10 (8)	73(63)
Nishikawa 2012^24^	77	PTX + S-1	Asian	53 (69)	67 (40–82)	77 (100)	0 (0)	0 (0)	77 (100)
	80	PTX + 5-FU		60 (75)	67 (47–90)	80 (100)	0 (0)	0 (0)	80 (100)
Sawaki 2009^25^	88	S-1	Asian	66 (75)	63 (32–77)	68 (77)	3 (3)	0 (0)	88 (100)
	89	5-FU/Lv		71 (80)	65 (44–77)	65 (73)	4 (4)	0 (0)	89 (100)
**S-1 vs Capecitabine**									
Kim 2012^8^	65	Ox + S-1	Asian	44 (68)	60 (28–77)	47 (72)	0 (0)	0 (0)	65 (100)
	64	Ox + Cap		45 (70)	61 (20–75)	46 (72)	0 (0)	0 (0)	64 (100)
Kobayashi 2015^9^	54	Cis + S-1	Asian	30 (55)	65 (44–74)	54 (100)	1 (2)	0 (0)	54 (100)
	55	Cis + Cap		45 (81)	65 (25–74)	55 (100)	2 (4)	0 (0)	55 (100)
Lee 2008^10^	45	S-1	Asian	37 (82)	71 (65–82)	45 (100)	2 (4)	NA	NA
	46	Cap		30 (65)	71 (66–78)	46 (100)	4 (9)	NA	NA

Abbreviations: 5-FU: 5-fluorouracil, Cap: capecitabine, Cis: cisplatin, DTX: docetaxel, Epi: Epirubicin, GEJ: gastro-esophageal junction, KPS: Karnofsky Performance Status, Lv: leucovorin, Ox: oxaliplatin, PTX: paclitaxel.

Notes: *This study also included patients with esophageal carcinoma. ^†^KPS ≤ 80 was given instead of WHO. ^††^Mean age was given instead of median age, performance status.

No major differences in study characteristics were observed (Table 1). The number of Western and Asian studies was five (n = 2652 patients, 56.3%) and ten (n = 2061 patients, 43.7%), respectively (Table 1). Seven (46.7%) studies were rated as low risk of bias for the primary outcome OS (Supplementary Figure 1A). Two (13.3%) and three (20.0%) studies were rated as unclear risk of bias on only one item or two items, respectively. The other three studies (20.0%) were rated as unclear on three items or were reported as abstract only. The risk of bias assessment for PFS is summarized in Supplementary Figure 1B.

### Efficacy

**Figure 3:**
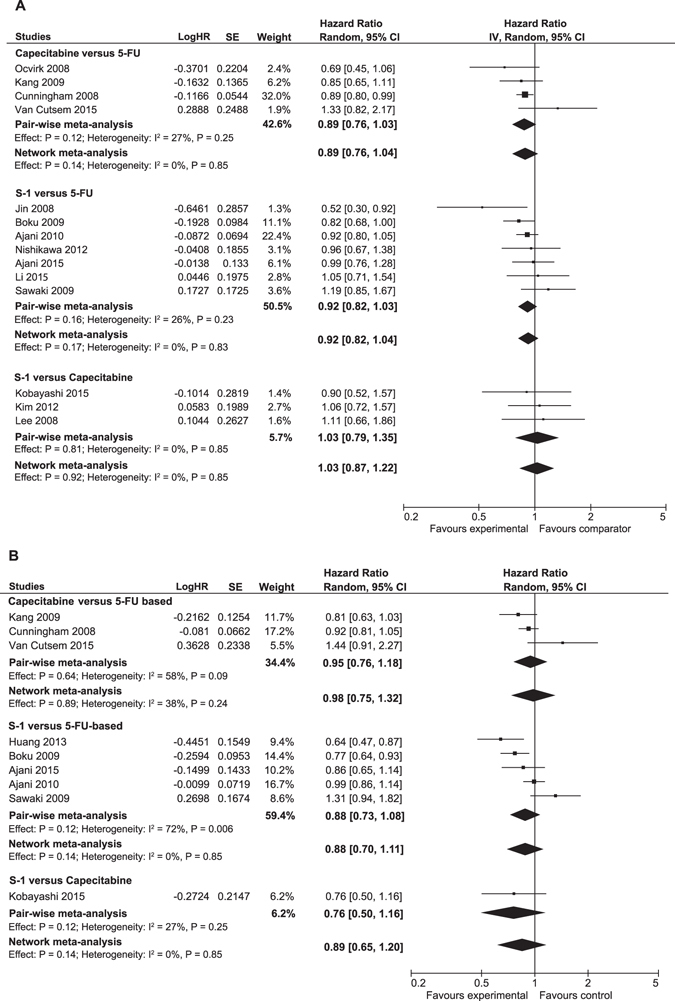
Results of the network meta-analysis for capecitabine, 5-FU and S-1 based regimens. (**A**) Forest plot of network meta-analysis for overall survival. (**B**) Forest plot for progression free survival. The lower pooled effect-size represents the combined hazard ratio and 95% Credible Intervals (95%CrI) derived from network-meta analysis. Abbreviations: 5-FU: 5-fluorouracil.

For OS, no significant differences were found by NMA for capecitabine-based (n = 798) compared to 5-FU-based regimens (n = 780), combined HR 0.89 (95%CrI 0.76–1.04), for S-1 based (n = 1353) compared to 5-FU-based regimens (n = 1224), combined HR 0.92 (95%CrI 0.82–1.04), and for S-1 based (n = 164) compared to capecitabine-based regimens (n = 165), combined HR 1.03 (95%CrI 0.87–1.22) (Fig. 3A). The results of the current NMA for S-1 based versus 5-FU-based and S-1-based versus capecitabine-based were in line with the results of the pair-wise meta-analysis (reported previously)^6^.

For the secondary outcome PFS, no significant differences were found by NMA for capecitabine-based (n = 753) compared to 5-FU-based regimens (n = 740), combined HR 0.98 (95%CrI 0.75–1.32), for S-1 based (n = 1201) compared to 5-FU-based regimens (n = 1063), combined HR 0.88 (95%CrI 0.70–1.11) and for S-1 based (n = 99) compared to capecitabine-based regimens (n = 101), combined HR 0.89 (0.95%CrI 0.65–1.20) (Fig. 3B). A statistically significant portion of heterogeneity was found for S-1 based compared to 5-FU-based regimens (I^2^ = 72.0%). No possible explanation of the heterogeneity was found by inspection of baseline characteristics of these studies (Table 1). Moreover, omitting studies one by one did neither result in substantial changes of the pooled HR nor in a reduction of the heterogeneity, indicated by the I^2^ (data not shown).

Sub-group NMA showed that the efficacy of regimens with capecitabine, S-1 and 5-FU in Asian and Western studies was in line with the overall results of the entire study population: no differences in Asian and Western patients were observed in terms of OS and PFS (Table 2).

**Table 2:** Network meta-analysis stratified by Asian and Western studies in overall survival and progression free survival.

	Overall survival		Progression free survival	
	Asian	Western	Asian	Western
Capecitabine vs 5-FU	0.84 (0.59–1.18)	0.92 (0.64–1.36)	0.82 (0.49–1.36)	1.06 (0.63–2.03)
S-1 vs 5-FU	0.90 (0.71–1.13)	0.94 (0.62–1.47)	0.84 (0.60–1.20)	0.94 (0.52–1.66)
S-1 vs capecitabine	1.08 (0.78–1.52)	1.02 (0.58–1.81)	1.03 (0.61–1.74)	1.00 (0.44–2.28)

Relative effects in combined hazard ratio and 95% Credible Intervals (95% CrI) derived from network-meta analysis for capecitabine, 5-FU and S-1 based cytotoxic regimens stratified by Asian studies and Western studies for overall survival and progression free survival. No significant differences were found among Asian and Western patients in efficacy between all fluoropyrimidines.

Abbreviations: 5-FU: 5-fluorouracil.

### Toxicity

**Figure 4:**
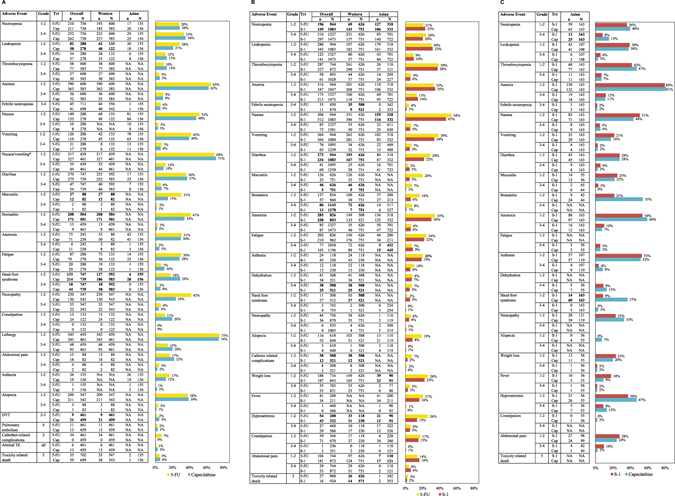
Toxicity of capecitabine, 5-FU and S-1 based regimens. (**A**) Toxicity of capecitabine-based compared to 5-FU-based regimens. (**B**) Toxicity of S-1 based compared to 5-FU-based regimens. (**C**) Toxicity of S-1 based compared to capecitabine-based regimens. The table shows the number of patients receiving that experienced grade 1-2 and grade 3-4 adverse events and the associated sample sizes for each treatment. The bar chart shows the percentage of both Western and Asian patients (overall) that experienced grade 1-2 and grade 3-4 adverse events and corresponds to the ‘overall’ column in the table. The number of adverse events that occurred in the Western subgroup or the Asian subgroup specifically are shown in the ‘Western’ and ‘Asian’ columns of the table. Bold indicates a statistically significant difference (P < 0.05) in a the occurrence of a given adverse event between two regimens determined by pair-wise meta-analysis. The number of adverse events for Western and Asian patients in Fig. 4B and C are a graphically representation of the data as published elsewhere^6^. However, in the current figures, additional adverse events were included, as well as the pooled number of adverse events of Western and Asian patients. Abbreviations: 5-FU: 5-fluorouracil, Cap: capecitabine, Trt: treatment.

In Fig. 4A,B and C, the occurrence of grade 1-2 and 3-4 AEs is shown for respectively capecitabine-based versus 5-FU-based, S-1 based versus 5-FU-based and S-1 based versus capecitabine-based regimens. Per comparison, we indicated if a specific AE occurred in both Asian and Western studies, Western studies only or Asian studies only.

For 5-FU-based compared to capecitabine-based regimens, also thrombo-embolic AE’s were extracted from a separate published article of the REAL-2 study^26^. Compared to 5-FU-based regimens, capecitabine-based regimens were associated with a higher rate of grade 1-2 and grade 3-4 hand-foot syndrome in both Asian and Western patients (Fig. 4A). In Western patients only, capecitabine-based regimens were associated with a higher rate of grade 2-3 deep venous thrombosis (DVT), but with a lower rate of grade 1-2 stomatitis and mucositis compared to 5-FU regimens. None of the adverse events showed a statistical significant difference between capecitabine-based and 5-FU-based regimens in Asian patients only.

S-1 based regimens were associated with a lower incidence of several grade 1-2 AEs and grade 3-4 dehydration compared to 5-FU-based regimens in both Asian and Western patients (Fig. 4B). In Western patients only, S-1 based regimens were associated with more grade 1-2 hand-foot syndrome, but with less catheter-related complications, and grade 3-4 mucositis, stomatitis, febrile neutropenia and toxicity-related-deaths compared to 5-FU-based regimens, as we described previously^6^. In Asian patients only, S-1 based regimens were associated with more grade 1-2 abdominal pain and grade 3-4 fatigue, but with less grade 1-2 nausea and weight loss compared to 5-FU-based regimens.

Finally, as Western studies have not been conducted to assess S-1 versus capecitabine therapy regimens, only three Asian studies reported toxicity for this comparison. A lower rate of grade 3-4 neutropenia and grade 1-2 hand-foot syndrome was found for S-1 based regimens compared to capecitabine-based regimens in Asian patients only (Fig. 4C).

### Publication bias

No evidence for publication bias was found in the Funnel plots for all comparisons and outcomes (Supplementary Figure 2).

## Discussion

This network meta-analysis of all available RCTs that have been conducted in the past decade provides a precise and cross-validated effect estimate of the fluoropyrimidines 5-FU, capecitabine and S-1 in the first-line treatment of advanced esophagogastric cancer. We showed that there is no difference in overall survival and progression-free-survival between 5-FU, capecitabine and S-1-based chemotherapy. In clinical practice, oral fluoropyrimidines capecitabine and S-1 are generally more convenient for patients compared to infusional 5-FU. On the other hand, for patients with dysphagia, which is common in gastro-oesophageal cancer, infusional 5-FU may be more suitable.

In addition to what was already known from previously conducted meta-analyses and pooled analyses^2, 13^, this NMA shows important new insights that are directly relevant for clinical practice. First, as there are only three small phase II RCTs in Asian populations that compared S-1 based and capecitabine-based regimens^8–10^, with the results derived from our indirect comparison we now can be more confident that S-1 based regimens have equal efficacy compared to capecitabine-based regimens in both Asian and Western patients. Combined with the other evidence for efficacy of S-1 based and 5-FU based regimens in Asian and Western patients presented in this NMA, we can conclude that the three fluoropyrimidines are equally effective in Western and Asian patients. This finding is important for both clinical practice and future RCTs, as practically all globally used first-line chemotherapy and targeted therapy regimens have fluoropyrimidine backbones^5^.

Second, we found that the fluoropyrimidines have a different toxicity profile in Asian patients, Western patients and in the population as a whole. Capecitabine-based regimens were associated with a higher incidence of clinically relevant adverse events hand-foot syndrome in Asian and Western patients and with DVT in Western patients compared to regimens including conventional 5-FU. However, there is a substantial risk of catheter-related (thrombo-embolic) complications with 5-FU, which is administrated by continuous venous infusion. Also, 5-FU based regimens are associated with a clinically relevant increased incidence of stomatitis and mucositis compared to regimens including oral fluoropyrimidines. On the other hand, S-1 based regimens were associated with less clinically relevant adverse events compared to regimens including conventional 5-FU, such as less febrile neutropenia and toxicity related deaths in Western patients and less grade 1-2 hand-foot syndrome compared to regimens including capecitabine in Asian patients. Although S-1 regimens also showed a lower rate of grade 3-4 hand-foot syndrome compared to capecitabine regimens (0% versus 3%), this difference did not reach statistical significance. From clinical perspective, occurrence of hand-foot syndrome is a very relevant parameter to monitor the well-being and quality of life of the patients receiving oral capecitabine. In a recently published phase III RCT in metastatic colorectal cancer, the occurrence of hand-foot syndrome, which was the primary endpoint, was significantly reduced in S-1 treated patients compared to capecitabine treated patients, without compromising efficacy^27^. These results are in line with our current findings for advanced esophagogastric cancer. The decision which fluoropyrimidine to prescribe for the individual patient should therefore be based on differences in toxicity profile.

We should also acknowledge some limitations of our work. This NMA was based on extracted summary data from published articles rather than on individual patient data. Second, many articles do not report adverse events that occurred less than a predefined percentage per study arm, which was most often a percentage of 5%. Although we provided a large overview of the toxicity profiles associated with these fluoropyrimidines in both Asian and Western patients, some adverse events may not have been included in the analyses. Third, for the analyses we assumed that the efficacy and toxicity of compounds that were identical in both arms of studies (i.e. cisplatin in the comparison S-1 plus cisplatin versus 5-FU plus cisplatin), was equal in both arms, so that the differences in efficacy and toxicity could be attributed to the fluoropyrimidines only (in this example: S-1 versus 5-FU). Due to slight differences in dosage (e.g. the dose of cisplatin in the FLAGS study^18^), effects cannot fully be attributed to differences in the fluoropyrimidines, but may be due to differences in the companion drug.

In conclusion, we found no difference in overall survival and progression-free-survival between 5-FU, capecitabine and S-1-based first-line chemotherapy for advanced esohagogastric cancer in both Asian and Western patients. The three fluoropyrimidines showed a different toxicity profile, and some relevant adverse events were observed with a lower frequency for S-1 based compared to 5-FU-based regimens (i.e. toxicity-related-death and febrile neutropenia) and capecitabine-based regimens (i.e. hand-foot syndrome).

## Material and Methods

### Protocol

The protocol was registered in PROSPERO with registration number: CRD42014015177.

### Literature search

We searched databases Medline, EMBASE and the Cochrane Central Register of Controlled Trials (CENTRAL) and meeting abstracts from the American Society of Clinical Oncology (ASCO) and European Society for Medical Oncology (ESMO) for randomized controlled trials up to January 2016. Medical subject headings (MeSH) and text words for esophagogastric cancer and for each treatment option, as described previously (Supplementary Table 1)^28^. The titles and abstracts were screened by NHM and MA. EtV and NHM screened the full articles. Disagreements were discussed with HvL until consensus was reached.

### Study selection

We included prospective phase II or III randomized controlled trials comparing first-line 5-FU, capecitabine or S-1 based chemotherapy regimens with equal backbones for previously untreated patients with pathologically proven metastatic, unresectable or recurrent adenocarcinoma of the stomach, esophagus, or GEJ.

### Data extraction and quality assessment

The primary outcome of our analyses was overall survival (OS). In addition, progression-free-survival (PFS), grade 1-2 (mild), and grade 3-4 (severe) adverse events (AE) were secondary outcomes. The Cochrane Risk of bias tool (version 5.1.0) was used to assess the quality of the included studies. Items were scored as low, high or unknown risk of bias.

### Statistical Analysis

Hazard ratios (HR) with 95% confidence intervals (95%CI) were extracted for time-to-event outcomes OS and PFS. Random effects pair-wise meta-analysis was conducted for direct comparisons simultaneously in R and Review Manager 5.3. For the NMA, studies were analysed in a 3-node network. Random-effects NMA was conducted using JAGS and the GeMTC package in R^29, 30^ (https://drugis.org/software/r-packages/gemtc). For the between studies heterogeneity and the relative treatment effects, vague priors were set. Four independent Markov chains were generated and ran for 5,000 adaptations and 20,000 inference iterations per chain to calculate the posterior distribution. Convergence of the Markov chains was assessed using Brooks-Gelman-Rubin diagnostic and the length of running time was extended if needed. HRs and 95% credible intervals (95%CrI) were calculated as effect-size to represent the combined direct and indirect relative treatment effects. In addition, the incidence of grade 1-2 and 3-4 adverse were compared using dichotomous pair-wise meta-analysis with Risk Ratios (RRs). In case of statistically significant heterogeneity, as determined by the Q-test, heterogeneity was explored by sensitivity analyses.

To evaluate differences in efficacy between fluoropyrimidines in Asian and Western patients, sub-group NMAs were conducted for studies in Asian and Western populations. Publication bias was assessed by funnel plots. All meta-analyses were performed using random-effects models and tested two-sided with α = 0.05.

## Electronic supplementary material

Supplementary information.
