# Supplementary material for: Capecitabine, 5-fluorouracil and S-1 based regimens for previously untreated advanced oesophagogastric cancer: A network meta-analysis
Source: Sci Rep. 2017 Aug 2;7:7142. doi: 10.1038/s41598-017-07750-3 (PMC5541083; doi:10.1038/s41598-017-07750-3)
Supplement: Supplementary file 1 — Supplementary information. [file 41598_2017_7750_MOESM1_ESM.pdf]

Supplementary content

Capecitabine, 5-fluorouracil and S-1 based regimens for previously untreated advanced oesophagogastric cancer: A network meta-analysis.

Emil ter Veer[1], Lok Lam Ngai[1], Gert van Valkenhoef[2], Nadia Haj Mohammad[1], Maarten C.J. Anderegg[3], Martijn G.H. van Oijen[1] and Hanneke W.M. van Laarhoven[1]*

Supplementary Figure 1. Risk of bias assessment

Risk of bias for overall survival (a). Risk of bias for progression free survival (b).

	Random sequence generation (selection bias)	Allocation concealment (selection bias)	Blinding of participants and personnel (performance bias)	Incomplete outcome data (attrition bias)	Selective reporting (reporting bias)	Other bias
Ajani 2010	?	?	+	+	+	+
Ajani 2015	?	?	+	+	+	?
Boku 2009	+	+	+	+	+	+
Cunningham 2008	+	+	+	+	+	+
Huang 2013	?	?	+	+	+	+
Jin 2008	+	+	+	+	+	?
Kang 2009	+	+	+	+	+	+
Kim 2012	+	+	+	+	+	+
Kobayashi 2015	?	?	+	+	+	?
Lee 2008	+	+	+	+	+	+
Li 2015	?	?	+	+	+	+
Nishikawa 2012	+	+	+	+	+	+
Ocvirk 2008	+	+	+	+	+	?
Sawaki 2009	?	?	+	+	+	?
Van Cutsem 2015	+	+	+	+	+	+

	Random sequence generation (selection bias)	Allocation concealment (selection bias)	Blinding of participants and personnel (performance bias)	Incomplete outcome data (attrition bias)	Selective reporting (reporting bias)	Other bias
Ajani 2010	?	?	+	+	+	+
Ajani 2015	?	?	?	+	+	?
Boku 2009	+	+	+	+	+	+
Cunningham 2008	+	+	?	+	+	+
Huang 2013	?	?	?	+	+	+
Jin 2008	+	+	+	+	+	?
Kang 2009	+	+	?	+	+	+
Kim 2012	+	+	?	+	+	+
Kobayashi 2015	?	?	?	+	+	?
Lee 2008	+	+	+	+	+	+
Li 2015	?	?	?	+	+	+
Nishikawa 2012	+	+	?	+	+	+
Ocvirk 2008	+	+	?	+	+	?
Sawaki 2009	?	?	?	+	+	?
Van Cutsem 2015	+	+	?	+	+	+

Supplementary Figure 2. Funnel plot for assessment of publication bias

Funnel plots for capecitabine versus 5-FU in OS (A) and PFS (B); S-1 versus 5-FU in OS (C) and PFS (D); S-1 versus capecitabine in OS (E) and PFS (F).

Supplementary Figure 2. Funnel plot for assessment of publication bias

Overall Survival

Progression Free Survival

A

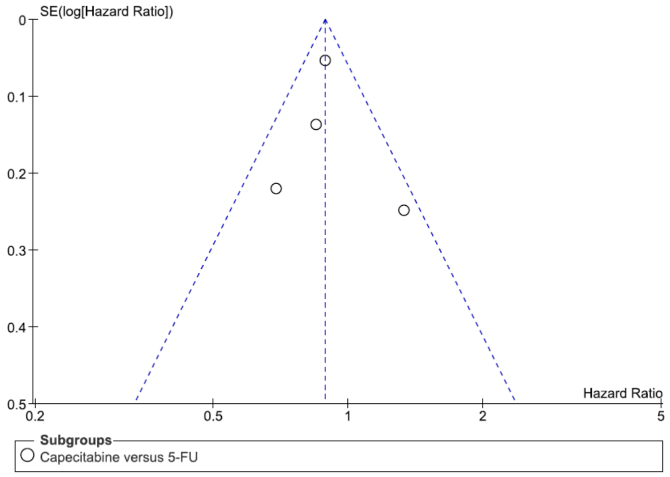

B

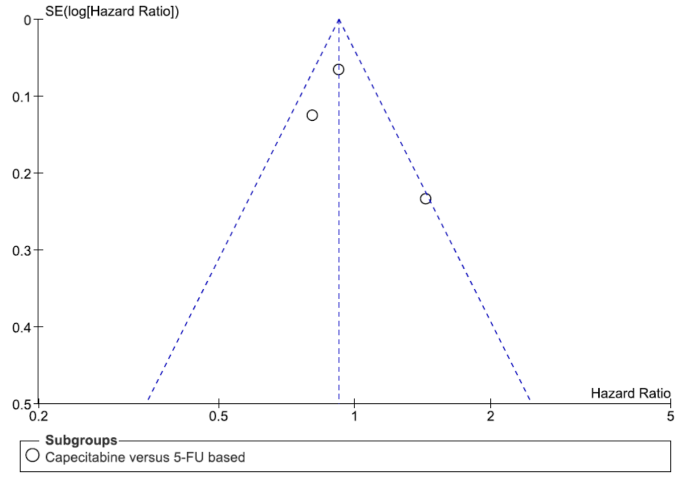

C

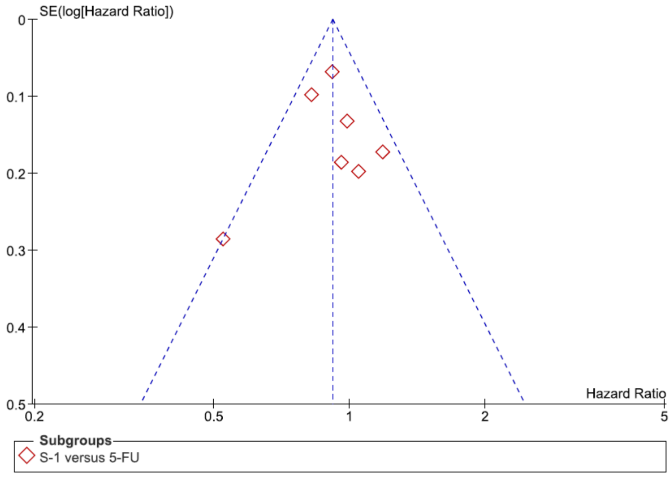

D

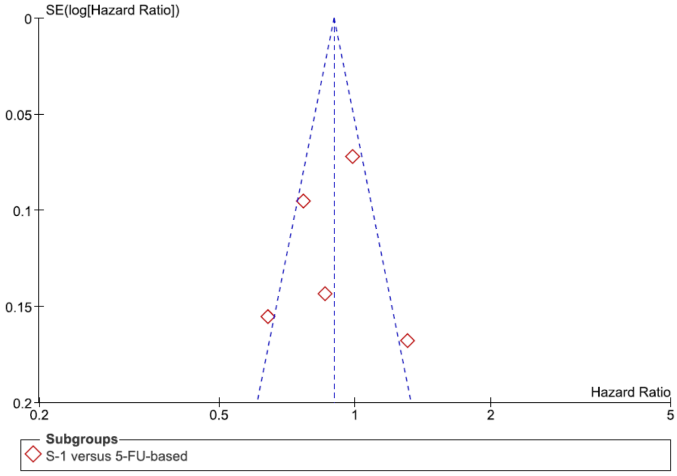

E

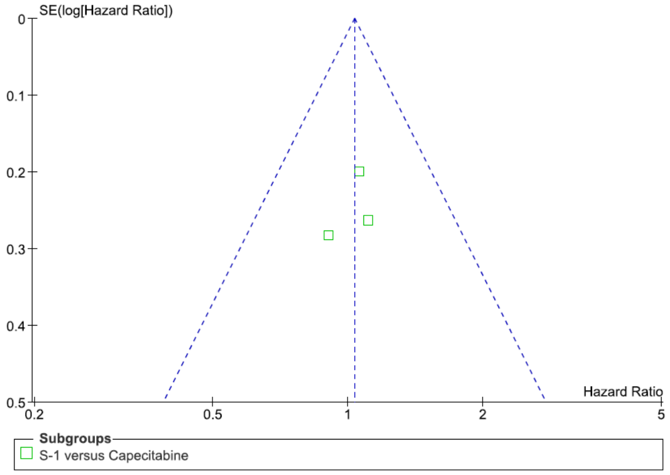

F

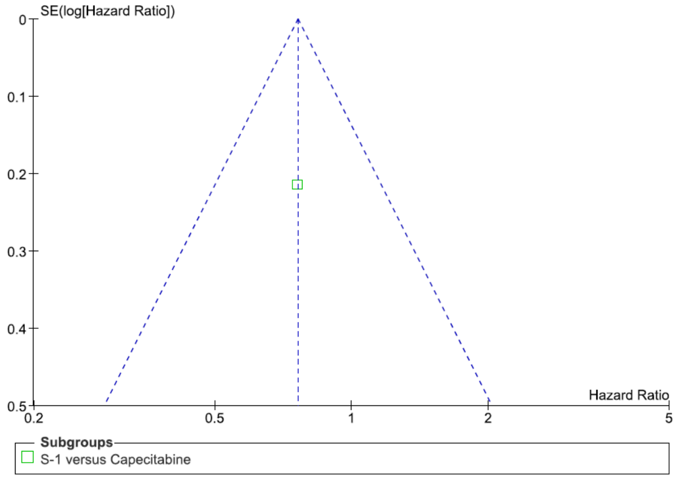

Supplementary Table 1. Search strategy

Cochrane Central Register of Controlled Trials (CENTRAL)

ID	Search
#1	MeSH descriptor: [Esophageal Neoplasms] explode all trees
#2	MeSH descriptor: [Stomach Neoplasms] explode all trees
#3	((esophag* or oesophag* or stomach or gastric or gastroesophag* or gastrooesophag*) and (neoplas* or cancer* or carcino* or adenocarcino* or tumor or tumors or tumour or tumours or malig*)):ti,ab,kw
#4	#1 or #2 or #3
#5	MeSH descriptor: [Palliative Care] explode all trees
#6	MeSH descriptor: [Neoplasm Metastasis] explode all trees
#7	(palliat* or advanced or metasta* or irresect* or unresect* or un-resect* or non-resect* or nonresect* or inopera* or non-opera* or nonopera* or unopera*):ti,ab,kw
#8	#5 or #6 or #7
#9	MeSH descriptor: [Drug Therapy, Combination] explode all trees
#10	MeSH descriptor: [Drug Combinations] explode all trees
#11	MeSH descriptor: [Antineoplastic Agents] explode all trees
#12	MeSH descriptor: [Anthracyclines] explode all trees
#13	MeSH descriptor: [Leucovorin] explode all trees
#14	MeSH descriptor: [Organoplatinum Compounds] explode all trees
#15	MeSH descriptor: [Oxonic Acid] explode all trees
#16	MeSH descriptor: [Taxoids] explode all trees
#17	(chemotherap* or polytherap* or polychemotherap* or combination* or two-agent* or two-drug* or double-drug* or doublet* or three-agent* or three-drug* or triple* or multi-agent or multi-drug or active agent* or antineoplastic* or anti-neoplastic* or anticancer* or anti-cancer* or antitumor* or anti-tumor* or antitumour* or anti-tumour* or anthracyclin* or capecitabine or carboplatin* or cisplatin* or docetaxel or doxorubicin* or epirubicin* or fluoropyrimidine* or fluorouracil or 5-FU or folinic acid or irinotecan or leucovorin* or mitomycin* or organoplatin* or oteracil or oxaliplatin* or oxonic acid or paclitaxel or platin* or S-1 or taxane* or tegafur):ti,ab,kw
#18	#9 or #10 or #11 or #12 or #13 or #14 or #15 or #16 or #17
#19	#4 and #8 and #18 in Trials

EMBASE via Ovid

1. esophagus tumor/ or exp esophagus cancer/
2. stomach tumor/ or exp stomach cancer/
3. ((esophag* or oesophag* or stomach or gastric or gastroesophag* or gastrooesophag*) adj5 (neoplas* or cancer* or carcino* or adenocarcino* or tumor or tumors or tumour or tumours or malig*)).ti,ab.
4. or/1-3
5. exp cancer palliative therapy/
6. exp metastasis/
7. advanced cancer/
8. inoperable cancer/
9. (palliat* or advanced or metasta* or irresect* or unresect* or un-resect* or non-resect* or nonresect* or inopera* or non-opera* or nonopera* or unopera*).ti,ab.
10. or/5-9
11. exp drug combination/

12. exp antineoplastic agent/
13. folinic acid/
14. platinum complex/
15. oteracil/
16. taxane derivative/
17. taxoid/
18. (chemotherap* or polytherap* or polychemotherap* or combination* or two-agent* or two-drug* or double-drug* or doublet* or three-agent* or three-drug* or triple* or multi-agent or multi-drug or active agent* or antineoplastic* or anti-neoplastic* or anticancer* or anti-cancer* or antitumor* or anti-tumor* or antitumour* or anti-tumour* or anthracyclin* or capecitabine or carboplatin* or cisplatin* or docetaxel or doxorubicin* or epirubicin* or fluoropyrimidine* or fluorouracil or 5-FU or folinic acid or irinotecan or leucovorin* or mitomycin* or organoplatin* or oteracil or oxaliplatin* or oxonic acid or paclitaxel or platin* or S-1 or taxane* or tegafur).ti,ab.
19. or/11-18
20. exp controlled clinical trial/ or randomized.ti,ab. or randomised.ti,ab. or placebo.ti,ab. or randomly.ti,ab. or trial.ti.
21. 4 and 10 and 19 and 20
22. limit 21 to (dutch or english)
23. limit 22 to (conference abstract or conference paper or "conference review" or conference proceeding)
24. 22 not 23

Medline via Pubmed

("Esophageal Neoplasms"[Mesh] OR "Stomach Neoplasms"[Mesh] OR ((esophag*[tiab] OR oesophag*[tiab] OR stomach[tiab] OR gastric[tiab] OR gastroesophag*[tiab] OR gastrooesophag*[tiab]) AND (neoplas*[tiab] OR cancer*[tiab] OR carcino*[tiab] OR adenocarcino*[tiab] OR tumor[tiab] OR tumors[tiab] OR tumour[tiab] OR tumours[tiab] OR malig*[tiab])))

AND

("Palliative Care"[Mesh] OR "Neoplasm Metastasis"[Mesh] OR palliat*[tiab] OR advanced[tiab] OR metasta*[tiab] OR irresect*[tiab] OR unresect*[tiab] OR un-resect*[tiab] OR non-resect*[tiab] OR nonresect*[tiab] OR inopera*[tiab] OR non-opera*[tiab] OR nonopera*[tiab] OR unopera*[tiab])

AND

("Drug Therapy, Combination"[Mesh] OR "Drug Combinations"[Mesh] OR "Antineoplastic Agents"[Mesh] OR "Antineoplastic Agents"[Pharmacological Action] OR "Anthracyclines"[Mesh] OR "Leucovorin"[Mesh] OR "Organoplatinum Compounds"[Mesh] OR "Oxonic Acid"[Mesh] OR "Taxoids"[Mesh] OR chemotherap*[tiab] OR polytherap*[tiab] OR polychemotherap*[tiab] OR combination*[tiab] OR two-agent*[tiab] OR two-drug*[tiab] OR double-drug*[tiab] OR doublet*[tiab] OR three-agent*[tiab] OR three-drug*[tiab] OR triple*[tiab] OR multi-agent[tiab] OR multi-drug[tiab] OR active agent*[tiab] OR antineoplastic*[tiab] OR anti-neoplastic*[tiab] OR anticancer*[tiab] OR anti-cancer*[tiab] OR antitumor*[tiab] OR anti-tumor*[tiab] OR antitumour*[tiab] OR anti-tumour*[tiab] OR anthracyclin*[tiab] OR capecitabine[tiab] OR carboplatin*[tiab] OR cisplatin*[tiab] OR docetaxel[tiab] OR doxorubicin*[tiab] OR epirubicin*[tiab] OR fluoropyrimidine*[tiab] OR fluorouracil[tiab] OR 5-FU[tiab] OR folinic acid[tiab] OR irinotecan[tiab] OR leucovorin*[tiab] OR mitomycin*[tiab] OR organoplatin*[tiab] OR

oteracil[tiab] OR oxaliplatin*[tiab] OR oxonic acid[tiab] OR paclitaxel[tiab] OR platin*[tiab] OR S-1[tiab] OR taxane*[tiab] OR tegafur[tiab]
AND
(randomized controlled trial[pt] OR controlled clinical trial[pt] OR randomized[tiab] OR randomised[tiab] OR placebo[tiab] OR clinical trials as topic[mesh:noexp] OR randomly[tiab] OR trial[ti]) AND (english[la] OR dutch[la])

Conference search: American Society of Clinical Oncology
Searching journal content for gastric (all words) in title or abstract and random* OR advance* OR metasta* (all words) in full text, from earliest publication date through January 2016

Conference search: European Society of Medical Oncology
Searching journal content for gastric (all words) in title or abstract and random* OR advance* OR metasta* (all words) in full text, from earliest publication date through January 2016
